# Report on Vaccination of the Twenty-First Regiment New York State Volunteers

**Published:** 1861-10

**Authors:** Charles H. Wilcox, Joseph A. Peters

**Affiliations:** Surgeon; Fort Runyon, Va.; Ass’t Surgeon; Fort Runyon, Va.


ART. IV.—*Report on Vaccination of the Twenty-First Regiment New York State Volunteers.*

FORT RUNYON, Va., July 27, 1861.

S. OAKLEY VANDERPOEL, M. D., *Surgeon General:*

Dear Sir:—I beg leave herewith to submit the following report of the vaccination of the Twenty-First Regiment New

York Volunteers, in accordance with General Orders No. 4, to the Medical Department.

*First*—There were vaccinated - - - - - - 706

*Second*—Those who showed evidences of previous vaccination were 644

*Third*—The number susceptible to the virus was - - 55

*Fourth*—Of those susceptible to the virus, forty-three (43) showed evidences of previous vaccination.

*Fifth*—The ages of those susceptible the second time are expressed in the following table:

| | |
|---|---|
| Between 18 and 20 years of age, - - - - - - - | 8 |
| " 20 " 25 " " - - - - - - | 23 |
| " 25 " 30 " " - - - - - - - | 6 |
| " 30 " 35 " " - - - - - | 1 |
| " 35 " 40 " " - - - - - - - | 4 |
| " 40 " 45 " " - - - - - | 1 |

Of the 706 vaccinated, 306 were vaccinated with *scale* virus, and 400 with the lymph on quills.

Of the 306 vaccinated with scale, 28 were susceptible to the virus, and 24 of them for the second time.

Of the 400 cases in which lymph was used 27 were susceptible, and 19 of them for the second time.

Of those previously vaccinated 167 had been vaccinated during the last seven years, and 477 previous to that time.

Of those previously vaccinated, who were again susceptible to the virus 11 had been vaccinated within 7 years, and 32 before that period.

It will be seen from the above facts that of the cases vaccinated with scale, 1 in 12.75 were susceptible the second time, and when lymph was used 1 in 21. It will also be seen that of the 167 vaccinated during the last 7 years, 1 in 15.18 was again susceptible, and among those vaccinated previous to that period 1 in 14.90.

Of those cases vaccinated with scale, many exhibited an areola of inflammation, enclosing a hard base, but no pustule; whereas in the cases in which lymph was used, there was no intermediate stage between an effect and a thorough working of the virus.

It should be noted that many cases were recorded as never having been vaccinated because no satisfactory evidence of the fact could be found, although they asserted that it had been done.

Two causes should not be lost sight of, which modify the result in all such cases as this, viz: the quality of the virus furnished, and the fact that some persons exhibit from infancy a marked want of susceptibility to the vaccine virus. Every practitioner meets such cases, of both infants and adults, upon whom no care in vaccination will produce the desired effect. Such cases we may have met, undoubtedly did meet, but as no re-vaccination was attempted, they cannot be accurately pointed out.

Great care was taken that no case should be put down as "susceptible to the virus" which did not exhibit the true pestule, no matter how severe inflammation may have attended the insertion of the virus, and it is believed that in this respect the figures given may be considered correct.

CHARLES H. WILCOX, Surgeon.
JOSEPH A. PETERS, Ass't Surgeon.

